# Supplementary material for: Functionally distinct cancer-associated fibroblast subpopulations establish a tumor promoting environment in squamous cell carcinoma
Source: Nat Commun. 2023 Sep 5;14:5413. doi: 10.1038/s41467-023-41141-9 (PMC10480447; doi:10.1038/s41467-023-41141-9)
Supplement: Supplementary file 1 — Supplementary Information [file 41467_2023_41141_MOESM1_ESM.pdf]

Supplementary Information

Functionally distinct cancer-associated fibroblast subpopulations establish a tumor promoting environment in squamous cell carcinoma

Sabrina Schütz[1], Llorenç Solé-Boldo[1], Carlota Lucena-Porcel[2,3], Jochen Hoffmann[4], Alexander Brobeil[2,3], Anke S. Lonsdorf[4], Manuel Rodríguez-Paredes[1*] & Frank Lyko[1*]

[1]Division of Epigenetics, DKFZ-ZMBH Alliance, German Cancer Research Center, 69120 Heidelberg, Germany; [2]Institute of Pathology, Ruprecht-Karls University of Heidelberg, 69120 Heidelberg, Germany; [3]Tissue Bank of the National Center for Tumor Diseases (NCT), 69120 Heidelberg, Germany; [4]Department of Dermatology, University Hospital, Ruprecht-Karls University of Heidelberg, 69120 Heidelberg, Germany

*Equal contribution.

Corresponding authors: Manuel Rodríguez-Paredes (m.rodriguez@dkfz.de, +49 6221424631)

Frank Lyko (f.lyko@dkfz.de, +49 6221423800)

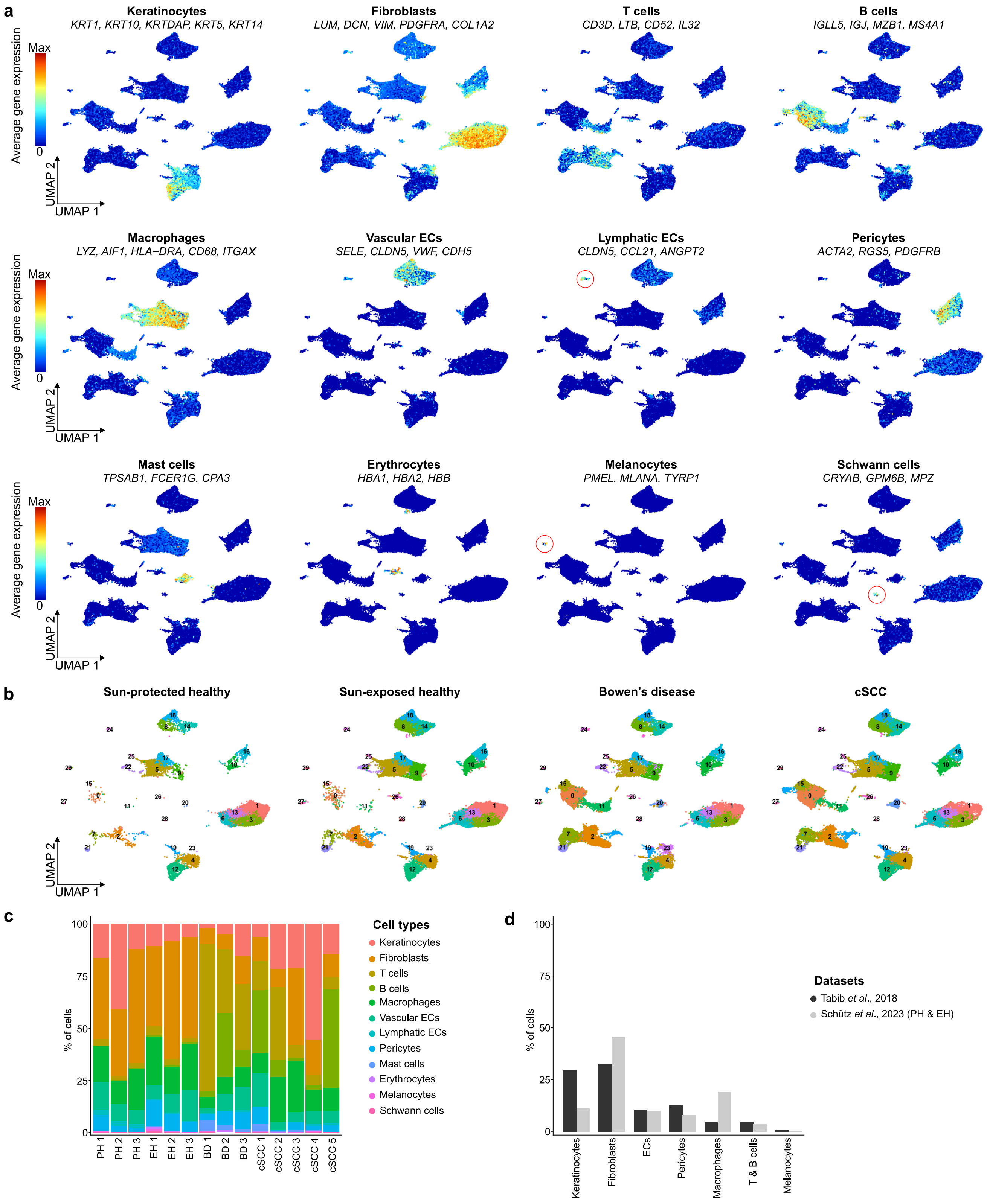
a
Keratinocytes
KRT1, KRT10, KRTDAP, KRT5, KRT14
Fibroblasts
LUM, DCN, VIM, PDGFRA, COL1A2
T cells
CD3D, LTB, CD52, IL32
B cells
IGLL5, IGJ, MZB1, MS4A1
Macrophages
LYZ, AIF1, HLA−DRA, CD68, ITGAX
Vascular ECs
SELE, CLDN5, VWF, CDH5
Lymphatic ECs
CLDN5, CCL21, ANGPT2
Pericytes
ACTA2, RGS5, PDGFRB
Mast cells
TPSAB1, FCER1G, CPA3
Erythrocytes
HBA1, HBA2, HBB
Melanocytes
PMEL, MLANA, TYRP1
Schwann cells
CRYAB, GPM6B, MPZ
Average gene expression
Max
0
UMAP 2
UMAP 1
b
Sun-protected healthy
Sun-exposed healthy
Bowen's disease
cSCC
c
% of cells
PH 1
PH 2
PH 3
EH 1
EH 2
EH 3
BD 1
BD 2
BD 3
cSCC 1
cSCC 2
cSCC 3
cSCC 4
cSCC 5
Cell types
Keratinocytes
Fibroblasts
T cells
B cells
Macrophages
Vascular ECs
Lymphatic ECs
Pericytes
Mast cells
Erythrocytes
Melanocytes
Schwann cells
d
% of cells
Keratinocytes
Fibroblasts
ECs
Pericytes
Macrophages
T & B cells
Melanocytes
Datasets
Tabib et al., 2018
Schütz et al., 2023 (PH & EH)

Supplementary Figure 1. scRNA-seq analysis identifies skin cell types. (a) UMAP plots visualizing the average gene expression of three to five known marker genes for typical cutaneous cell types. Red indicates maximum gene expression, while blue indicates low or no expression. **(b)** UMAP plots showing the integrated dataset with all clusters for UVR-protected healthy skin, chronically UVR-exposed healthy skin, BD and cSCC samples separately. **(c)** Bar plot depicting cell type proportions for all scRNA-seq samples. **(d)** Bar plot showing cell type proportions of the human skin scRNA-seq study from Tabib *et al.*, 2018[1] and the present study. UVR: ultraviolet radiation, PH: UVR-protected healthy, EH: UVR-exposed healthy, BD: Bowen's disease, cSCC: cutaneous squamous cell carcinoma. Source data are provided as a Source Data file.

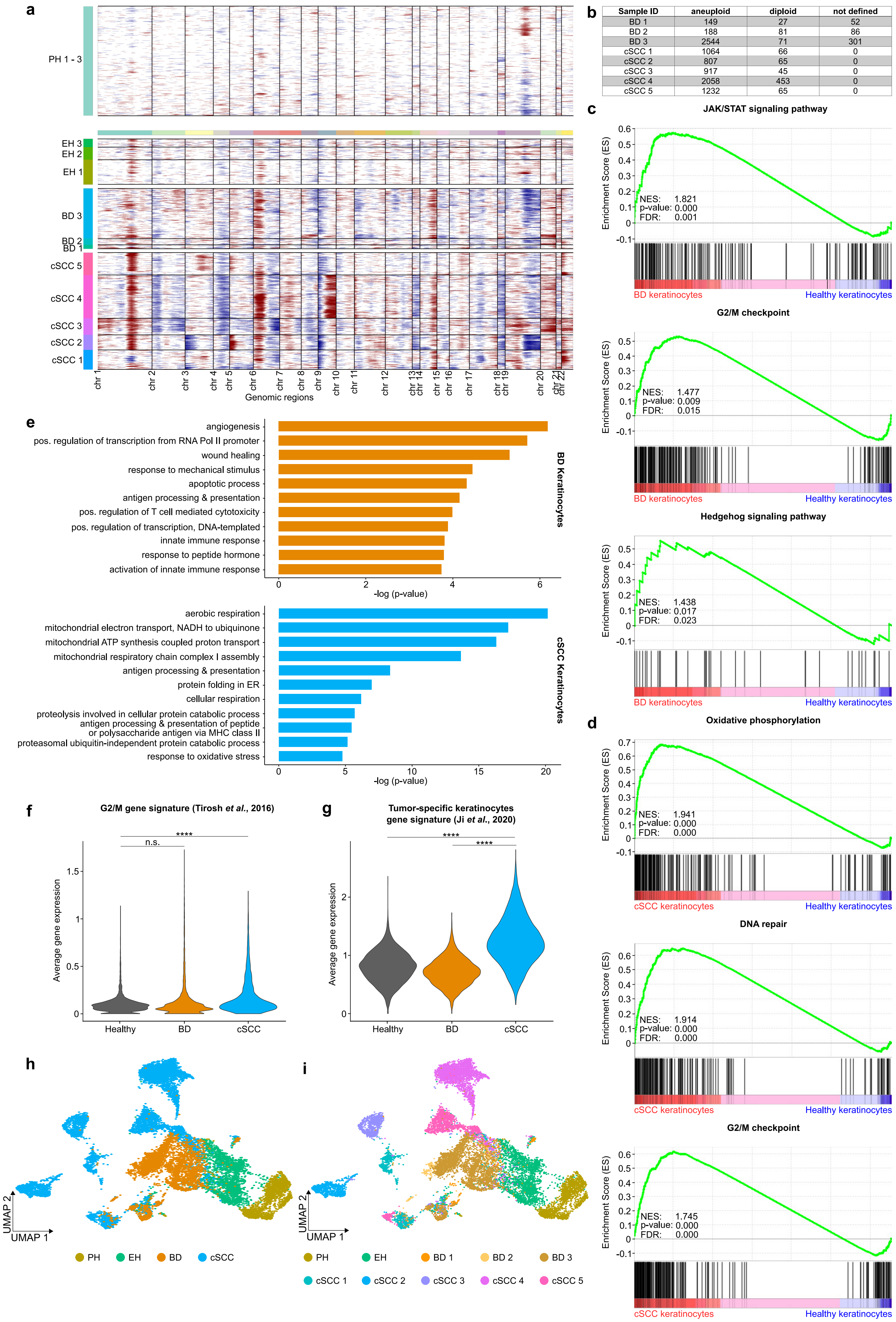

Sample ID	aneuploid	diploid	not defined
BD 1	149	27	52
BD 2	188	81	86
BD 3	2544	71	301
cSCC 1	1064	66	0
cSCC 2	807	65	0
cSCC 3	917	45	0
cSCC 4	2058	453	0
cSCC 5	1232	65	0

Supplementary Figure 2. Characterization of malignant keratinocytes derived from BD and cSCC samples. (a) Heatmaps visualizing the distribution of copy number variation (CNV) events in keratinocytes from all scRNA-seq samples across the chromosomes. Each row represents a single cell and columns correspond to genes, ordered by chromosome position. Gene amplifications are indicated in red and deletions are marked in blue. **(b)** Table including the total numbers of keratinocytes from BD and cSCC samples that were either classified as aneuploid or diploid, and non-defined keratinocytes. **(c) & (d)** Gene Set Enrichment Analysis (GSEA) plots for different pro-tumorigenic pathways that are activated in BD keratinocytes **(c)** or cSCC keratinocytes **(d)** compared to healthy keratinocytes. Normalized Enrichment Scores (NES), false discovery rates (FDR) and *p*-values are given for all plots, based on the Kolmogorov Smirnov test. **(e)** Top 11 terms from Gene Ontology (GO) analyses performed with the most upregulated genes in BD keratinocytes or cSCC keratinocytes compared to healthy keratinocytes. Terms are ordered according to *p*-value determined by the Fisher's Exact test. **(f)** Violin plot displaying the average gene expression of proliferation-related genes important in the G2/M cell cycle phase[2] in keratinocytes from healthy samples, BD and cSCC. Healthy vs. BD: *p*-value = 0.76, Healthy vs. cSCC: *p*-value < 2.2e-16. **(g)** Violin plot showing the average gene expression of a publicly available cSCC-derived keratinocyte gene signature[3] in keratinocytes from healthy samples, BD and cSCC. Healthy vs. cSCC: *p*-value < 2.2e-16, BD vs. cSCC: *p*-value < 2.2e-16. **(h)** UMAP plot displaying sample type-specific clusters of keratinocytes after merging, but not integrating, all datasets. **(i)** UMAP plot showing patient-specific clusters of keratinocytes from BD and cSCC samples after merging, but not integrating, all datasets. For violin plots, statistical analyses were performed using a two-sided Wilcoxon Rank Sum test (****: *p*-value < 0.0001). chr: chromosome, UVR: ultraviolet radiation, PH: UVR-protected healthy, EH: UVR-exposed healthy, BD: Bowen's disease, cSCC: cutaneous squamous cell carcinoma, n.s.: not significant. Source data are provided as a Source Data file.

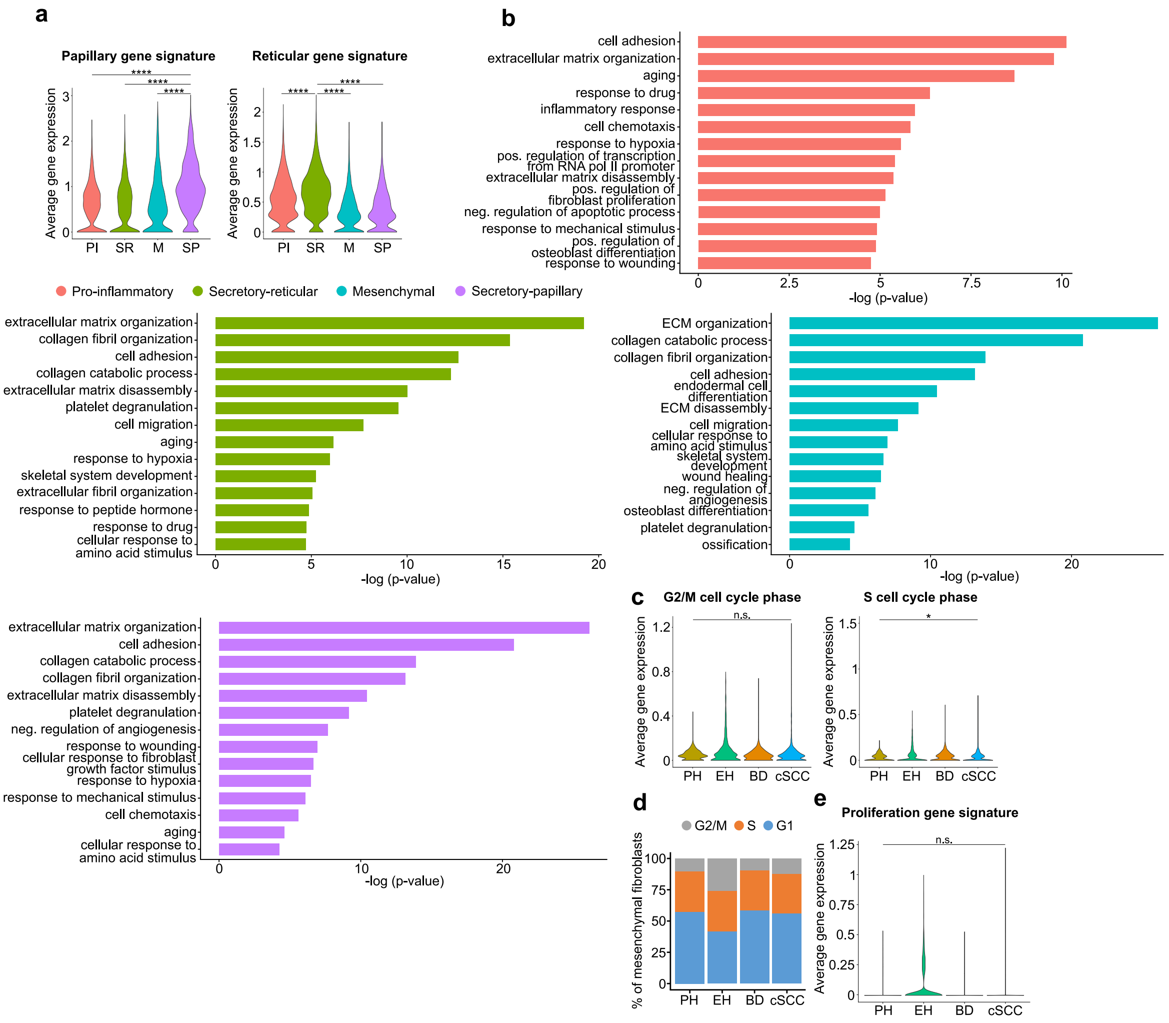

a
Papillary gene signature
Reticular gene signature
Average gene expression
PI SR M SP
Pro-inflammatory
Secretory-reticular
Mesenchymal
Secretory-papillary
b
cell adhesion
extracellular matrix organization
aging
response to drug
inflammatory response
cell chemotaxis
response to hypoxia
pos. regulation of transcription from RNA pol II promoter
extracellular matrix disassembly
pos. regulation of fibroblast proliferation
neg. regulation of apoptotic process
response to mechanical stimulus
pos. regulation of osteoblast differentiation
response to wounding
-log (p-value)
extracellular matrix organization
collagen fibril organization
cell adhesion
collagen catabolic process
extracellular matrix disassembly
platelet degranulation
cell migration
aging
response to hypoxia
skeletal system development
extracellular fibril organization
response to peptide hormone
response to drug
cellular response to amino acid stimulus
-log (p-value)
ECM organization
collagen catabolic process
collagen fibril organization
cell adhesion
endodermal cell differentiation
ECM disassembly
cell migration
cellular response to amino acid stimulus
skeletal system development
wound healing
neg. regulation of angiogenesis
osteoblast differentiation
platelet degranulation
ossification
-log (p-value)
extracellular matrix organization
cell adhesion
collagen catabolic process
collagen fibril organization
extracellular matrix disassembly
platelet degranulation
neg. regulation of angiogenesis
response to wounding
cellular response to fibroblast growth factor stimulus
response to hypoxia
response to mechanical stimulus
cell chemotaxis
aging
cellular response to amino acid stimulus
-log (p-value)
c
G2/M cell cycle phase
S cell cycle phase
Average gene expression
n.s.
PH EH BD cSCC
d
G2/M S G1
% of mesenchymal fibroblasts
PH EH BD cSCC
e
Proliferation gene signature
Average gene expression
n.s.
PH EH BD cSCC

Supplementary Figure 3. Previously defined fibroblast subpopulations are maintained during cSCC development. (a) Violin plots showing the average gene expression of known marker genes for papillary and reticular fibroblasts respectively[4] in all fibroblasts analyzed. All indicated comparisons: *p*-value < 2.2e-16. **(b)** Top 14 terms from Gene Ontology (GO) analyses performed with the most representative genes expressed in each fibroblast subpopulation. Terms are ordered according to *p*-value, determined by the Fisher's Exact test. **(c)** Violin plots visualizing the average gene expression of cell cycle-related genes[2] in mesenchymal fibroblasts from all sample types. G2/M phase PH vs. cSCC: *p*-value = 0.399, S phase PH vs. cSCC: *p*-value = 0.0271. **(d)** Bar plot depicting cell cycle phase ratios for mesenchymal fibroblasts detected in the different sample types. **(e)** Violin plot showing the average gene expression of known genes related to cell proliferation[5] for mesenchymal fibroblasts from all sample types. PH vs. cSCC: *p*-value = 0.879. For violin plots, statistical analyses were performed using a two-sided Wilcoxon Rank Sum test (*: *p*-value < 0.05, ****: *p*-value < 0.0001). PH: UVR-protected healthy, EH: UVR-exposed healthy, BD: Bowen's disease, cSCC: cutaneous squamous cell carcinoma, PI: pro-inflammatory, SR: secretory-reticular, M: mesenchymal, SP: secretory-papillary, n.s.: not significant. Source data are provided as a Source Data file.

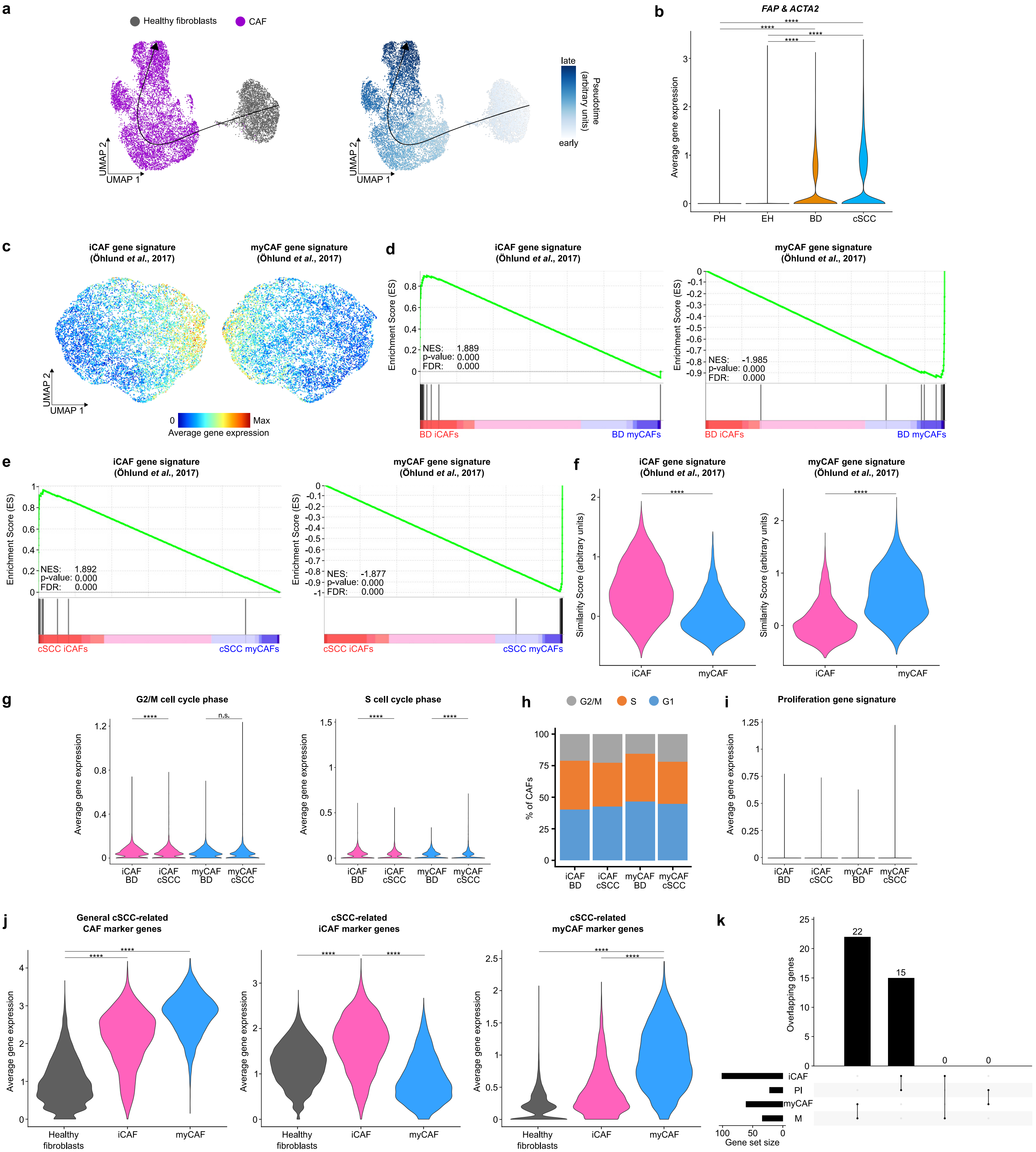

a
Healthy fibroblasts
CAF
UMAP 1
UMAP 2
late
early
Pseudotime (arbitrary units)
b
FAP & ACTA2
Average gene expression
PH
EH
BD
cSCC
c
iCAF gene signature (Öhlund et al., 2017)
myCAF gene signature (Öhlund et al., 2017)
0
Max
Average gene expression
d
Enrichment Score (ES)
NES: 1.889
p-value: 0.000
FDR: 0.000
NES: -1.985
p-value: 0.000
FDR: 0.000
BD iCAFs
BD myCAFs
e
NES: 1.892
p-value: 0.000
FDR: 0.000
NES: -1.877
p-value: 0.000
FDR: 0.000
cSCC iCAFs
cSCC myCAFs
f
Similarity Score (arbitrary units)
iCAF
myCAF
g
G2/M cell cycle phase
S cell cycle phase
n.s.
iCAF BD
iCAF cSCC
myCAF BD
myCAF cSCC
h
G2/M
S
G1
% of CAFs
i
Proliferation gene signature
j
General cSCC-related CAF marker genes
cSCC-related iCAF marker genes
cSCC-related myCAF marker genes
Healthy fibroblasts
k
Overlapping genes
22
15
0
0
PI
M
Gene set size

Supplementary Figure 4. Detection of iCAFs and myCAFs in BD and cSCC. (a) UMAP plots visualizing the progression trajectory of healthy fibroblasts from UVR-protected skin towards CAFs from BD and cSCC samples. White and light blue indicate an earlier time point during this process, while dark blue indicates later time points on an arbitrary pseudotime axis. **(b)** Violin plot showing the average gene expression of *FAP* and *ACTA2* in fibroblasts from UVR-protected healthy and chronically UVR-exposed healthy skin samples as well as BD and cSCC. All indicated comparisons: p-value < 2.2e-16. **(c)** UMAP plots depicting the average gene expression of pancreatic iCAF- and myCAF-related gene signatures[6, 7]. Red indicates maximum gene expression, while blue indicates low or no expression. **(d) & (e)** Gene Set Enrichment Analysis (GSEA) plots for gene signatures of pancreatic iCAFs and myCAFs[6, 7] that are enriched in BD- **(d)** and cSCC-derived **(e)** iCAFs and myCAFs, respectively. For all plots, the normalized Enrichment Score (NES), false discovery rate (FDR) and p-value are given, based on the Kolmogorov Smirnov test. **(f)** Violin plots visualizing similarity scores in arbitrary units between BD/cSCC-derived CAFs and gene signatures of pancreatic iCAFs and myCAFs[6, 7]. Both comparisons: p-value < 2.2e-16. **(g)** Violin plots showing the average gene expression of cell cycle-related genes[2] in iCAFs and myCAFs from BD and cSCC. G2/M phase iCAF BD vs. iCAF cSCC: p-value = 6.63e-05, myCAF BD vs. myCAF cSCC: p-value = 0.1, S phase iCAF BD vs. iCAF cSCC: p-value = 2.81e-09, myCAF BD vs. myCAF cSCC: p-value = 3.31e-05. **(h)** Bar plot depicting cell cycle phase ratios for iCAFs and myCAFs, in BD and cSCC. **(i)** Violin plot visualizing the average gene expression of known genes related to cell proliferation[5] for iCAFs and myCAFs from BD and cSCC. **(j)** Violin plots showing the average gene expression of general CAF marker genes and specific iCAF/myCAF marker genes defined in cSCC (Supplementary Data 4) for healthy fibroblasts (PH & EH) and CAFs (BD & cSCC). All indicated comparisons: p-value = 0. **(k)** Bar plot depicting the overlap of the most representative genes of iCAFs, myCAFs, pro-inflammatory fibroblasts and mesenchymal fibroblasts. For violin plots, statistical analyses were performed using a two-sided Wilcoxon Rank Sum test (****: p-value < 0.0001). UVR: ultraviolet radiation, PH: UVR-protected healthy, EH: UVR-exposed healthy, BD: Bowen's disease, cSCC: cutaneous squamous cell carcinoma,

iCAF: inflammatory cancer-associated fibroblast, myCAF: myofibroblastic cancer-associated fibroblast, PI: pro-inflammatory, M: mesenchymal, n.s.: not significant. Source data are provided as a Source Data file.

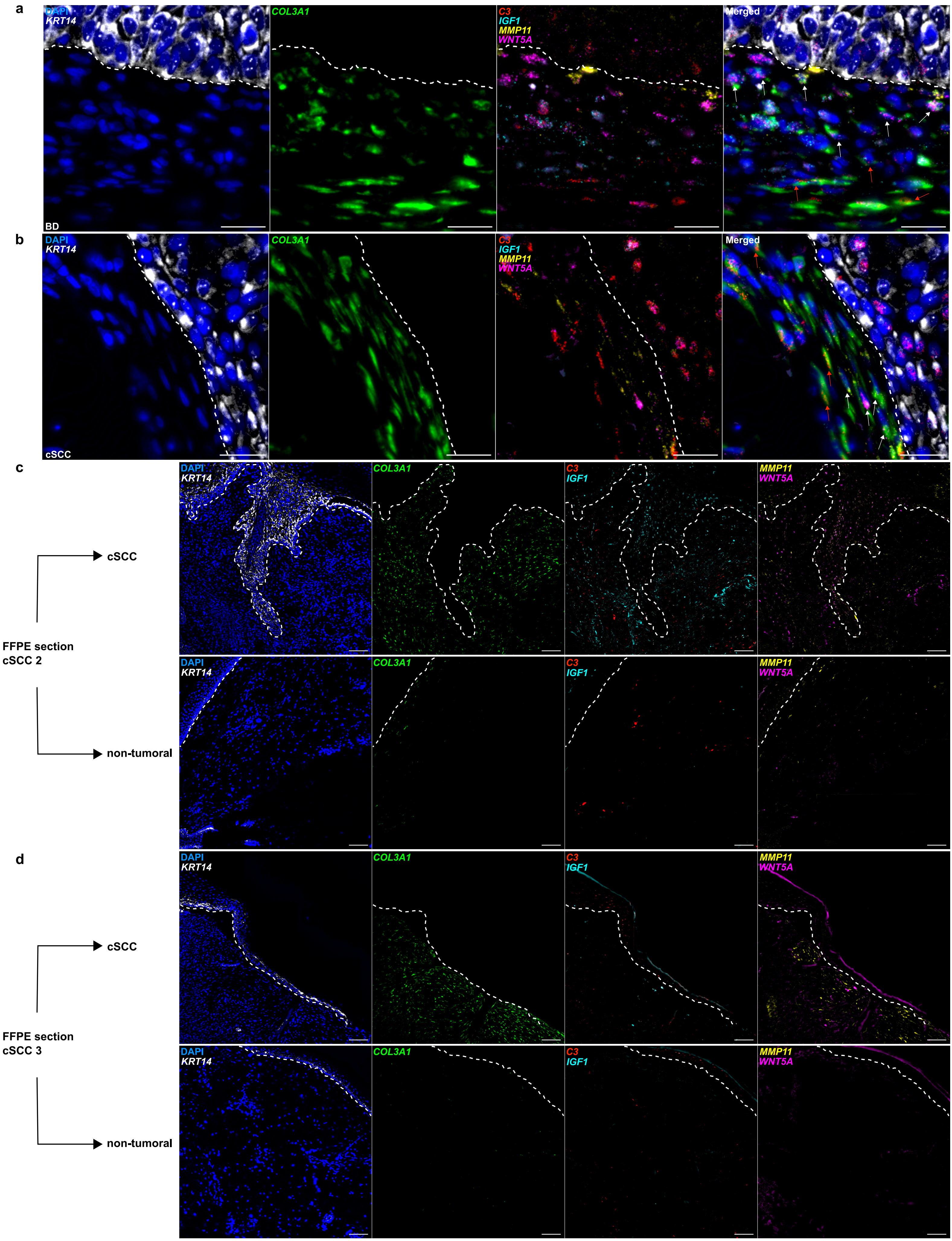

a
DAPI
KRT14
BD
COL3A1
C3
IGF1
MMP11
WNT5A
Merged
b
DAPI
KRT14
cSCC
COL3A1
C3
IGF1
MMP11
WNT5A
Merged
c
FFPE section
cSCC 2
cSCC
non-tumoral
DAPI
KRT14
COL3A1
C3
IGF1
MMP11
WNT5A
d
FFPE section
cSCC 3
cSCC
non-tumoral
DAPI
KRT14
COL3A1
C3
IGF1
MMP11
WNT5A

Supplementary Figure 5. Multiplexed RNA FISH detects iCAFs and myCAFs in BD and cSCC but not in adjacent non-tumoral skin. Exemplary microscopy images of FFPE BD **(a)** and cSCC **(b)** samples as well as a direct comparison of a tumor area and a non-tumoral but chronically UVR-exposed skin area of the same FFPE sections cSCC 2 **(c)** and cSCC 3 **(d)**. All images show the respective channels visualizing the hybridization with fluorescent probes against the mRNA of *KRT14* (white) as keratinocyte marker, *COL3A1* (green) as general CAF marker, *C3* (red) and *IGF1* (cyan) as iCAF markers, as well as *MMP11* (yellow) and *WNT5A* (magenta) as myCAF markers. Nuclei were counterstained with DAPI. Dashed lines separate epidermal keratinocytes from dermal cells. Scale bars: 25µm for (a) and (b) or 100µm for (c) and (d). White arrows indicate myCAFs whereas red arrows indicate iCAFs. BD: n = 3 patients, cSCC: n = 3 patients. FISH: fluorescence *in situ* hybridization, UVR: ultraviolet radiation, FFPE: formalin-fixed paraffin embedded, iCAF: inflammatory cancer-associated fibroblast, myCAF: myofibroblastic cancer-associated fibroblast, BD: Bowen's disease, cSCC: cutaneous squamous cell carcinoma, EH: UVR-exposed healthy skin.

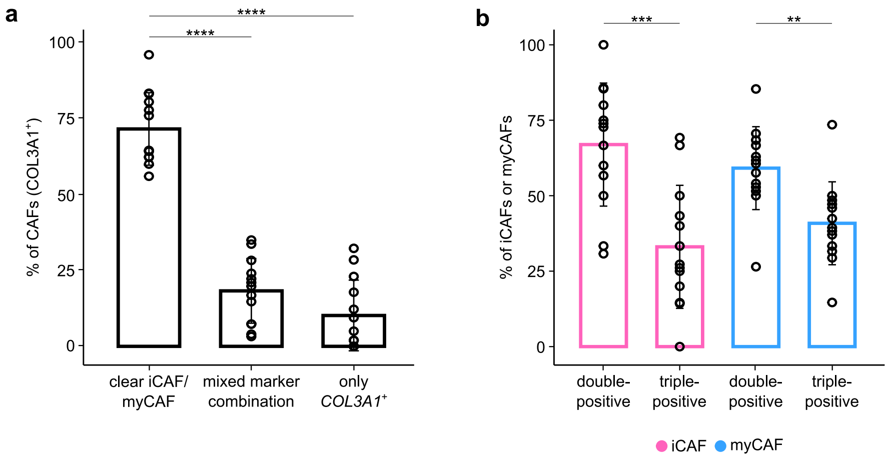

a
b
****
****
100
75
50
25
0
% of CAFs (COL3A1⁺)
clear iCAF/ myCAF
mixed marker combination
only COL3A1⁺
***
**
% of iCAFs or myCAFs
double-positive
triple-positive
double-positive
triple-positive
iCAF
myCAF

Supplementary Figure 6. Conditions for quantification of multiplexed RNA FISH assays. **(a)** Bar plot showing the proportions of CAFs (*COL3A1*$^{+}$) that were unambiguously classified as iCAFs or myCAFs in cSCC due to the expression of either *C3* and/or *IGF1*, or *MMP11* and/or *WNT5A*, respectively. CAFs showing mixed combinations for iCAF and myCAF marker genes, as well as cells expressing only *COL3A1* but no specific iCAF/myCAF markers, were not considered for following analyses and quantifications. Clear iCAFs/myCAFs vs. mixed marker: *p*-value = 1.215e-011, clear iCAFs/myCAFs vs. only *COL3A1*$^{+}$: *p*-value = 1.487e-012. **(b)** Bar plot visualizing the ratios of all iCAFs and myCAFs detected in cSCC with respect to the expression of marker gene combinations. Double-positive cells showed co-expression of *COL3A1* as general CAF marker and one subpopulation-specific gene (*C3* or *IGF1* for iCAFs, and *MMP11* or *WNT5A* for myCAFs). Triple-positive cells showed co-expression of *COL3A1* and both of the respective subpopulation-specific genes. iCAFs double vs. triple: *p*-value = 0.000288, myCAFs double vs. triple: *p*-value = 0.00240. Statistical analyses were performed using two-sided t-tests (**: *p*-value < 0.01, ***: *p*-value < 0.001, ****: *p*-value < 0.0001) and error bars show the standard deviation. Bars indicate mean values and each dot represents a dermal region with around 100 *COL3A1*$^{+}$ cells/CAFs (cSCC: n = 13 dermal regions). Source data are provided as a Source Data file.

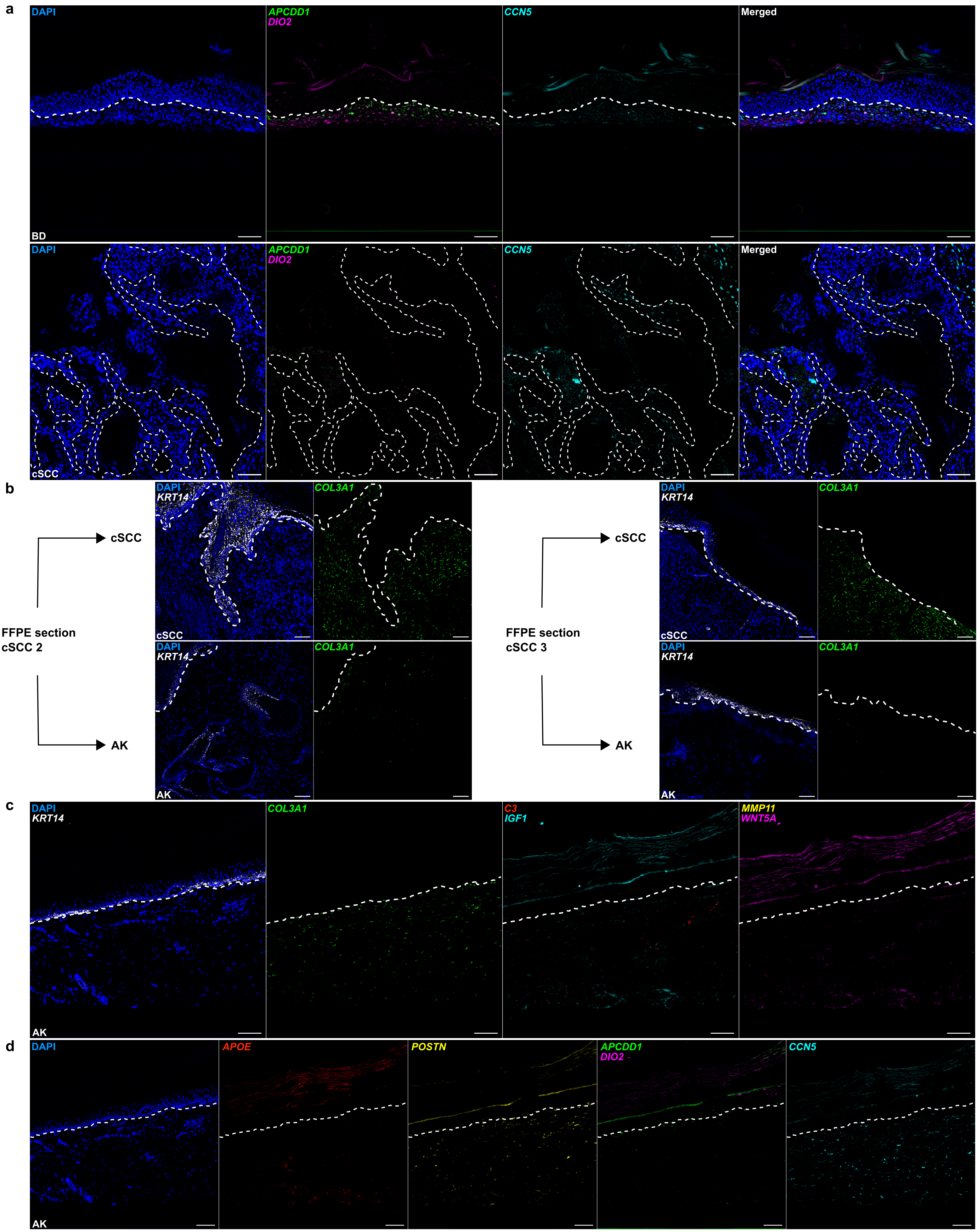

a
DAPI
APCDD1
DIO2
CCN5
Merged
BD
cSCC
b
FFPE section
cSCC 2
cSCC
AK
KRT14
COL3A1
FFPE section
cSCC 3
c
C3
IGF1
MMP11
WNT5A
d
APOE
POSTN

Supplementary Figure 7. Multiplexed RNA FISH detects fibroblast subpopulation marker genes in the cSCC disease continuum. (a) Representative microscopy images of FFPE BD and cSCC samples hybridized with fluorescent probes against the mRNA of *APCDD1* (green) and *DIO2* (magenta) as secretory-papillary fibroblast marker genes, and *CCN5* (cyan) as secretory-reticular marker. Nuclei were counterstained with DAPI. Dashed lines separate epidermal keratinocytes from dermal cells. Scale bar: 100µm. **(b)** Illustrative microscopy images of tumor areas and AK regions in the same FFPE sections cSCC 2 and cSCC 3 hybridized with fluorescent probes against the mRNA of *KRT14* (white) as keratinocyte marker and *COL3A1* (green) as general cSCC-CAF marker. Nuclei were counterstained with DAPI. Dashed lines separate epidermal keratinocytes from dermal cells. Scale bar: 100µm. **(c)** Exemplary microscopy image of a FFPE AK sample hybridized with fluorescent probes against the mRNA of *KRT14* (white) as keratinocyte marker, *COL3A1* (green) as general CAF marker, *C3* (red) and *IGF1* (cyan) as iCAF markers as well as *MMP11* (yellow) and *WNT5A* (magenta) as myCAF markers. Nuclei were counterstained with DAPI. Dashed lines separate epidermal keratinocytes from dermal cells. Scale bar: 100µm. **(d)** Representative microscopy image of a FFPE AK sample hybridized with fluorescent probes against the mRNA of *APOE* (red) as pro-inflammatory fibroblast marker, *POSTN* (yellow) as mesenchymal fibroblast marker, *APCDD1* (green) and *DIO2* (magenta) as secretory-papillary fibroblast marker genes as well as *CCN5* (cyan) as secretory-reticular marker. Nuclei were counterstained with DAPI. Dashed lines separate epidermal keratinocytes from dermal cells. Scale bar: 100µm. AK: n = 3 patients, BD: n = 3 patients, cSCC: n = 3 patients. FISH: fluorescence *in situ* hybridization, FFPE: formalin-fixed paraffin embedded, iCAF: inflammatory cancer-associated fibroblast, myCAF: myofibroblastic cancer-associated fibroblast, AK: actinic keratosis, BD: Bowen's disease, cSCC: cutaneous squamous cell carcinoma.

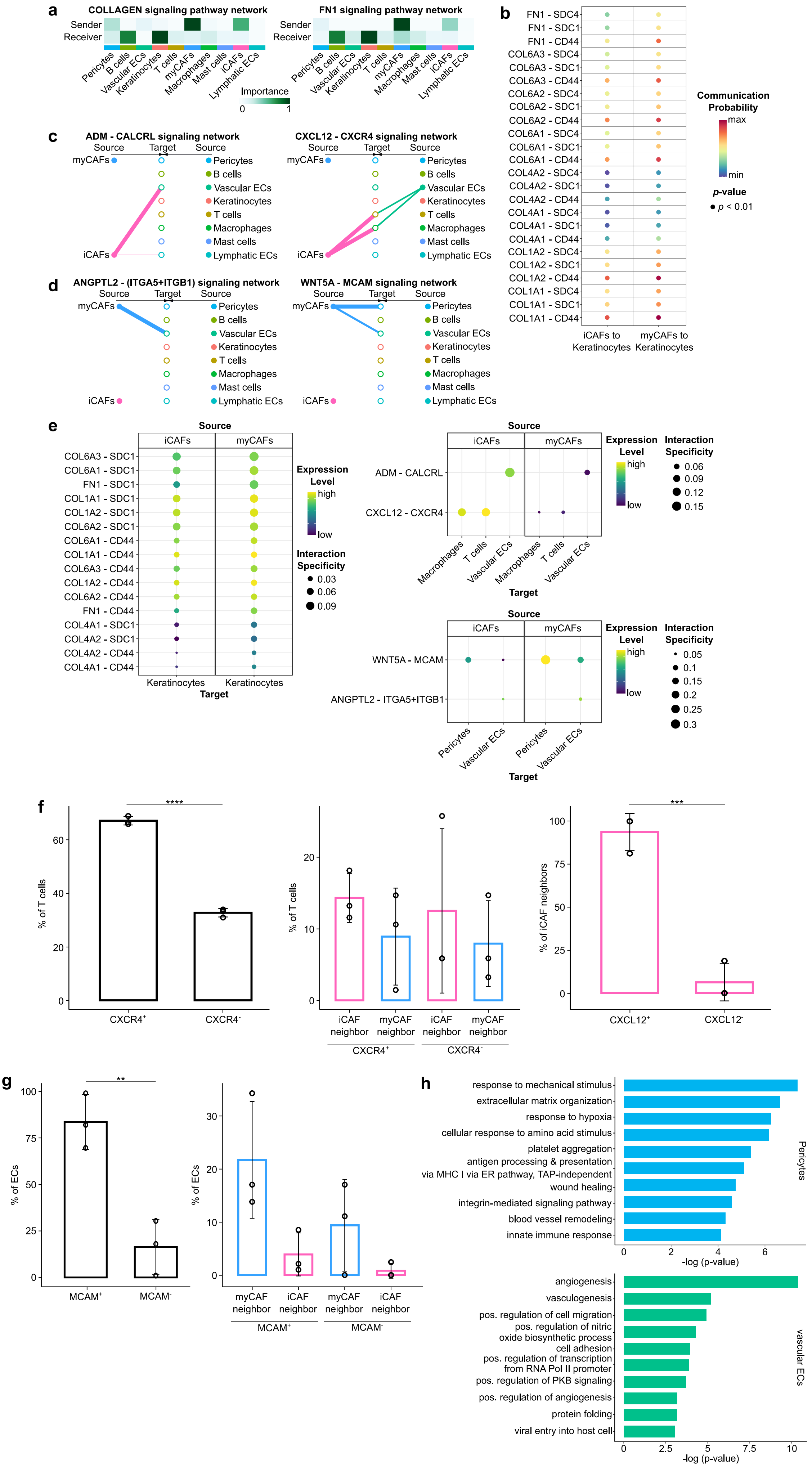
a
COLLAGEN signaling pathway network
FN1 signaling pathway network
Sender
Receiver
Pericytes
B cells
Vascular ECs
Keratinocytes
T cells
myCAFs
Macrophages
Mast cells
iCAFs
Lymphatic ECs
Importance
0
1
b
FN1 - SDC4
FN1 - SDC1
FN1 - CD44
COL6A3 - SDC4
COL6A3 - SDC1
COL6A3 - CD44
COL6A2 - SDC4
COL6A2 - SDC1
COL6A2 - CD44
COL6A1 - SDC4
COL6A1 - SDC1
COL6A1 - CD44
COL4A2 - SDC4
COL4A2 - SDC1
COL4A2 - CD44
COL4A1 - SDC4
COL4A1 - SDC1
COL4A1 - CD44
COL1A2 - SDC4
COL1A2 - SDC1
COL1A2 - CD44
COL1A1 - SDC4
COL1A1 - SDC1
COL1A1 - CD44
iCAFs to Keratinocytes
myCAFs to Keratinocytes
Communication Probability
max
min
p-value
p < 0.01
c
ADM - CALCRL signaling network
CXCL12 - CXCR4 signaling network
Source
Target
Source
myCAFs
iCAFs
d
ANGPTL2 - (ITGA5+ITGB1) signaling network
WNT5A - MCAM signaling network
e
Source
iCAFs
myCAFs
COL6A3 - SDC1
COL6A1 - SDC1
FN1 - SDC1
COL1A1 - SDC1
COL1A2 - SDC1
COL6A2 - SDC1
COL6A1 - CD44
COL1A1 - CD44
COL6A3 - CD44
COL1A2 - CD44
COL6A2 - CD44
FN1 - CD44
COL4A1 - SDC1
COL4A2 - SDC1
COL4A2 - CD44
COL4A1 - CD44
Keratinocytes
Target
Expression Level
high
low
Interaction Specificity
0.03
0.06
0.09
ADM - CALCRL
CXCL12 - CXCR4
0.06
0.09
0.12
0.15
WNT5A - MCAM
ANGPTL2 - ITGA5+ITGB1
0.05
0.1
0.15
0.2
0.25
0.3
f
****
% of T cells
CXCR4+
CXCR4-
iCAF neighbor
myCAF neighbor
***
% of iCAF neighbors
CXCL12+
CXCL12-
g
**
% of ECs
MCAM+
MCAM-
h
response to mechanical stimulus
extracellular matrix organization
response to hypoxia
cellular response to amino acid stimulus
platelet aggregation
antigen processing & presentation via MHC I via ER pathway, TAP-independent
wound healing
integrin-mediated signaling pathway
blood vessel remodeling
innate immune response
Pericytes
-log (p-value)
angiogenesis
vasculogenesis
pos. regulation of cell migration
pos. regulation of nitric oxide biosynthetic process
cell adhesion
pos. regulation of transcription from RNA Pol II promoter
pos. regulation of PKB signaling
pos. regulation of angiogenesis
protein folding
viral entry into host cell
vascular ECs
-log (p-value)

Supplementary Figure 8. CAFs affect cancer cells and tumor microenvironment via common and specific signaling pathways. (a) Heatmaps visualizing predictions (CellChat) of the most important "sender" and "receiver" cell types within the cSCC tissue for different signaling pathway networks. White color or light green correlate with no or low involvement, while dark green represents high importance. **(b)** Dot plot showing significant interactions (CellChat) between CAF-derived ligands and receptors expressed in malignant keratinocytes in cSCC. Red indicates high interaction probability and blue represents low interaction probability. The dot size correlates with the *p*-value of the interaction, based on the method trimean. **(c)** Plots depicting signaling networks (CellChat) with iCAFs as the most important "sender" cells secreting the ligands ADM (left) and CXCL12 (right). Line thickness correlates with interaction strength. Thicker lines indicate stronger signals. **(d)** Plots visualizing signaling networks (CellChat) with myCAFs as the most important "sender" cells secreting the ligands ANGPTL2 (left) and WNT5A (right). **(e)** Dot plots showing significant (p-value < 0.01) interactions (LIANA) between CAF-derived ligands and receptors expressed in keratinocytes or different non-malignant cell types in cSCC, based on the robust rank aggregation method. Purple color correlates with low expression levels and yellow indicates high expression levels. Interaction specificity is depicted by the dot size. **(f)** Bar plots visualizing signal quantification for iCAF interactions in RNA FISH assays with respect to receptor expression (*CXCR4*) in T cells (p-value = 0.0000115, left), their iCAF/myCAF proximity (center), and ligand expression (*CXCL12*) in iCAFs (p-value = 0.000584, right). **(g)** Bar plots depicting signal quantification for myCAF interactions in RNA FISH assays with respect to receptor expression (*MCAM*) in vascular ECs (p-value = 0.00508, left), and their iCAF/myCAF proximity with myCAFs expressing the ligand *WNT5A* (right). **(h)** Top 10 terms from Gene Ontology (GO) analyses with the most upregulated genes from chronically UVR-exposed healthy skin to cSCC tumors in pericytes and vascular ECs. Terms are ordered according to *p*-value, determined by the Fisher's Exact test. For quantifications in (f) and (g), statistical analyses were performed using two-sided t-tests (**: p-value < 0.01, ***: p-value < 0.001, ****: p-value < 0.0001) and error bars show the standard deviation. Bars indicate mean values and each dot represents a dermal

region with around 100 T cells or vascular ECs (cSCC: n = 3 dermal regions). UVR: ultraviolet radiation, iCAF: inflammatory cancer-associated fibroblast, myCAF: myofibroblastic cancer-associated fibroblast, EC: endothelial cell, cSCC: cutaneous squamous cell carcinoma. Source data are provided as a Source Data file.

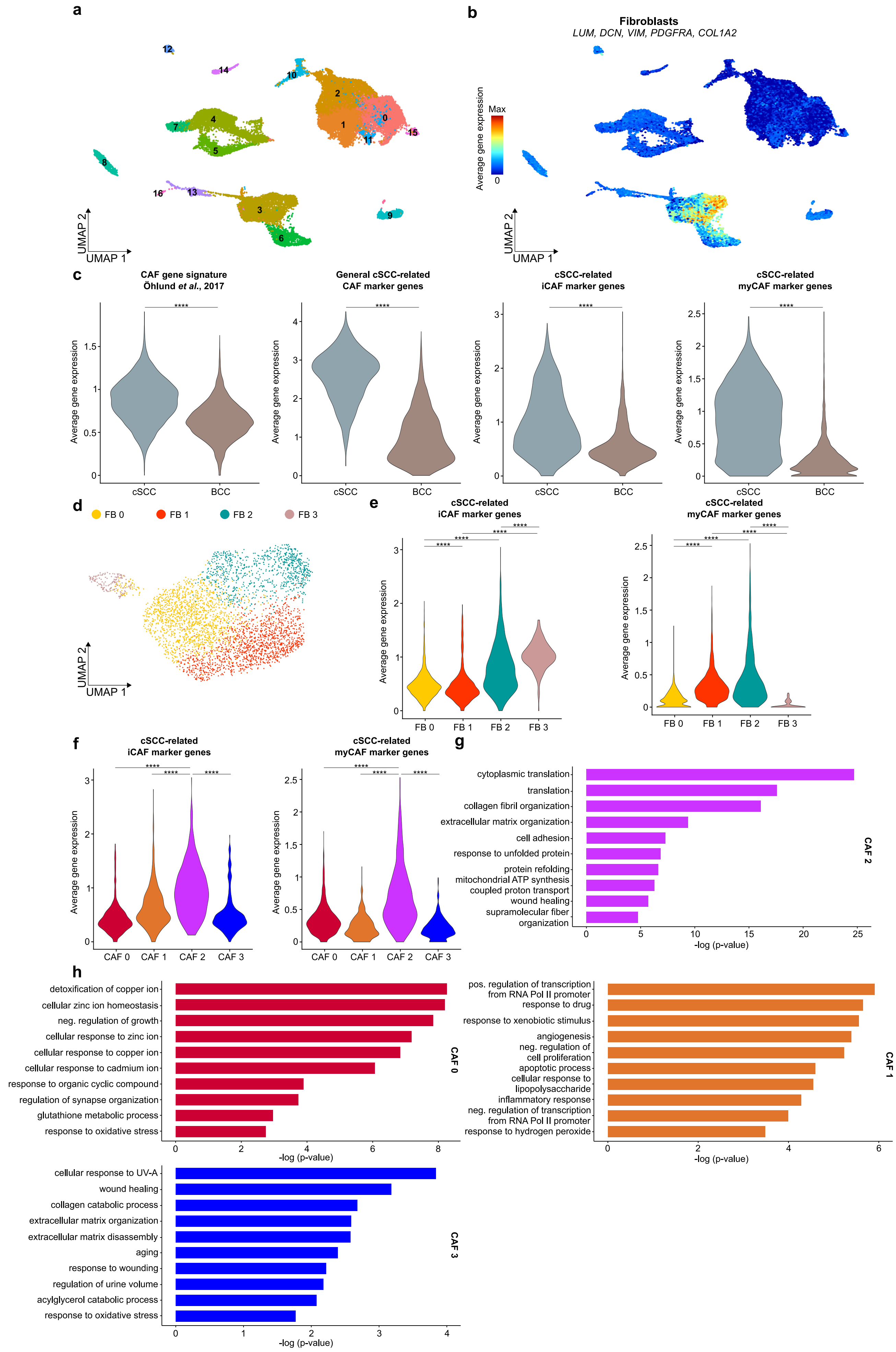
a
b
Fibroblasts
LUM, DCN, VIM, PDGFRA, COL1A2
Average gene expression
Max
0
UMAP 2
UMAP 1
c
CAF gene signature
Öhlund et al., 2017
General cSCC-related
CAF marker genes
cSCC-related
iCAF marker genes
cSCC-related
myCAF marker genes
cSCC
BCC
d
FB 0
FB 1
FB 2
FB 3
e
f
CAF 0
CAF 1
CAF 2
CAF 3
g
cytoplasmic translation
translation
collagen fibril organization
extracellular matrix organization
cell adhesion
response to unfolded protein
protein refolding
mitochondrial ATP synthesis
coupled proton transport
wound healing
supramolecular fiber
organization
-log (p-value)
h
detoxification of copper ion
cellular zinc ion homeostasis
neg. regulation of growth
cellular response to zinc ion
cellular response to copper ion
cellular response to cadmium ion
response to organic cyclic compound
regulation of synapse organization
glutathione metabolic process
response to oxidative stress
pos. regulation of transcription
from RNA Pol II promoter
response to drug
response to xenobiotic stimulus
angiogenesis
neg. regulation of
cell proliferation
apoptotic process
cellular response to
lipopolysaccharide
inflammatory response
neg. regulation of transcription
from RNA Pol II promoter
response to hydrogen peroxide
cellular response to UV-A
wound healing
collagen catabolic process
extracellular matrix organization
extracellular matrix disassembly
aging
response to wounding
regulation of urine volume
acylglycerol catabolic process
response to oxidative stress

Supplementary Figure 9. Assessment of cSCC-related iCAF and myCAF marker gene expression in BCC CAFs. (a) UMAP plot visualizing unsupervised clustering of nine BCC samples from Yerly *et al.*, 2022[8] and Guerrero-Juarez *et al.*, 2022[9]. **(b)** UMAP plot depicting the average gene expression of known fibroblast marker genes. Red indicates maximum gene expression, while blue indicates low or no expression. **(c)** Violin plots displaying the average gene expression levels of previously described PDAC-related CAF signatures[6], as well as our newly defined cSCC-related CAF signatures in cSCC-derived CAFs and BCC-derived fibroblasts. All indicated comparisons: *p*-value < 2.2e-16. **(d)** UMAP plot of re-clustered fibroblasts from all BCC samples. **(e)** Violin plots showing the average gene expression of cSCC-related iCAF (FB 0 vs. FB 1: *p*-value = 9.09e-07, FB 0 vs. FB 2: *p*-value < 2.2e-16, FB 1 vs. FB 3: *p*-value < 2.2e-16, FB 2 vs. FB 3: *p*-value = 3.46e-15, left) and myCAF (all indicated comparisons: *p*-value < 2.2e-16, right) marker genes in BCC fibroblasts. **(f)** Violin plots showing the average gene expression of cSCC-related iCAF (left) and myCAF (right) marker genes in BCC CAFs. All indicated comparisons: *p*-value < 2.2e-16. **(g)** Top 10 terms from a Gene Ontology (GO) analysis with the most representative genes of BCC CAF cluster 2. Terms are ordered according to *p*-value, determined by the Fisher's Exact test. **(h)** Top 10 terms from GO analyses with the most representative genes of BCC CAF clusters 0, 1 and 3. Terms are ordered according to *p*-value, determined by the Fisher's Exact test. For violin plots, statistical analyses were performed using a two-sided Wilcoxon Rank Sum test (****: *p*-value < 0.0001). FB: fibroblast, iCAF: inflammatory cancer-associated fibroblast, myCAF: myofibroblastic cancer-associated fibroblast, cSCC: cutaneous squamous cell carcinoma, BCC: basal cell carcinoma, PDAC: pancreatic ductal adenocarcinoma. Source data are provided as a Source Data file.

Sample ID	Gender	Age (years)	Localization	Chronic UVR exposure	Immune status	Diagnosis	Grading & tumor thickness	Number of cells
PH 1*	male	53 – 70	inguinal	no	IC	healthy	N/A	2,592
PH 2*	male		inguinal	no	IC	healthy	N/A	1,833
PH 3*	male		inguinal	no	IC	healthy	N/A	4,386
EH 1	male	47 – 87	temple	yes	IC	healthy	N/A	13,439
EH 2	male		cheek	yes	IC	healthy	N/A	8,757
EH 3	male		head	yes	IC	healthy	N/A	7,380
BD 1	male	65 – 85	head	yes	IC	BD	N/A	10,047
BD 2**	male		upper arm	yes	IC	BD	early invasive	7,183
BD 3**	male		shoulder	yes	IC	BD	early invasive	18,988
cSCC 1	male	72 – 93	head	yes	IC	cSCC	G3-4 / 6.0 mm	18,336
cSCC 2	male		head	yes	IC	cSCC	G2 / 3.6 mm	4,054
cSCC 3	male		head	yes	IC	cSCC	G2 / 6.0 mm	4,533
cSCC 4	male		head	yes	IC	cSCC	G1 / 8.0 mm	4,536
cSCC 5	male		head	yes	IC	cSCC	G1-2 / 3.2 mm	8,989

Supplementary Table 1. Overview of the samples used for single-cell RNA sequencing.

UVR: ultraviolet radiation, PH: UVR-protected healthy, EH: chronically UVR-exposed healthy, BD: Bowen's disease, cSCC: cutaneous squamous cell carcinoma, IC: immunocompetent, * raw data obtained from Solé-Boldo *et al.*, 2020[10], ** samples obtained from the same patient.

Sample type	Total fibroblast number	PI fibroblasts	SR fibroblasts	M fibroblasts	SP fibroblasts
PH	3,982	1,547	1,437	376	622
EH	13,637	5,998	3,557	2,285	1,797
BD	3,807	1,279	1,152	764	612
cSCC	5,956	1,318	1,236	2,455	947

Supplementary Table 2. Total numbers of fibroblasts and their subpopulations in each sample type analyzed.

PH: UVR-protected healthy, EH: chronically UVR-exposed healthy, BD: Bowen's disease, cSCC: cutaneous squamous cell carcinoma, PI: pro-inflammatory, SR: secretory-reticular, M: mesenchymal, SP: secretory-papillary.

Sample ID	Gender	Age (years)	Localization	Immune status	Grading & tumor thickness	Biopsy
EH 1	male	65 – 83	face	IC	N/A	incisional
EH 2	male		head	IC	N/A	incisional
EH 3	male		face	IC	N/A	incisional
AK 1	male	82 – 88	head	IC	N/A	incisional
AK 2	male		face	IC	N/A	shave
AK 3	male		head	IC	N/A	shave
BD 1	male	77 – 82	head	IC	N/A	shave
BD 2	male		head	IC	N/A	shave
BD 3	male		head	IC	N/A	shave
cSCC 1	male	71 – 85	head	IC	G2 / 4.0 mm	incisional
cSCC 2	male		head	IC	G1 / 2.0 mm	incisional
cSCC 3	male		head	IC	G2 / 1.2 mm	incisional
cSCC 4	male		face	IC	G2 / 5.0 mm	incisional
cSCC 5	male		face	IC	G2 / 3.6 mm	incisional

Supplementary Table 3. Overview of the FFPE samples used for RNA FISH.

EH: chronically UVR-exposed healthy, UVR: ultraviolet radiation, AK: actinic keratosis, BD: Bowen's disease, cSCC: cutaneous squamous cell carcinoma, IC: immunocompetent.

References

1. Tabib, T., Morse, C., Wang, T., Chen, W., Lafyatis, R. SFRP2/DPP4 and FMO1/LSP1 Define Major Fibroblast Populations in Human Skin. *J. Invest. Dermatol.* **138**, 802-810 (2018).
2. Tirosh, I. et al. Dissecting the multicellular ecosystem of metastatic melanoma by single-cell RNA-seq. *Science* **352**, 189-196 (2016).
3. Ji, A. L. et al. Multimodal Analysis of Composition and Spatial Architecture in Human Squamous Cell Carcinoma. *Cell* **182**, 497-514.e422 (2020).
4. Solé-Boldo, L. et al. Single-cell transcriptomes of the human skin reveal age-related loss of fibroblast priming. *Commun. Biol.* **3**, 188 (2020).
5. Whitfield, M. L., George, L. K., Grant, G. D., Perou, C. M. Common markers of proliferation. *Nat. Rev. Cancer* **6**, 99-106 (2006).
6. Öhlund, D. et al. Distinct populations of inflammatory fibroblasts and myofibroblasts in pancreatic cancer. *J. Exp. Med.* **214**, 579-596 (2017).
7. Elyada, E. et al. Cross-Species Single-Cell Analysis of Pancreatic Ductal Adenocarcinoma Reveals Antigen-Presenting Cancer-Associated Fibroblasts. *Cancer Discov.* **9**, 1102-1123 (2019).
8. Yerly, L. et al. Integrated multi-omics reveals cellular and molecular interactions governing the invasive niche of basal cell carcinoma. *Nat. Commun.* **13**, 4897 (2022).
9. Guerrero-Juarez, C. F. et al. Single-cell analysis of human basal cell carcinoma reveals novel regulators of tumor growth and the tumor microenvironment. *Sci. Adv.* **8**, eabm7981 (2022).
10. Solé-Boldo, L. et al. Single-cell transcriptomes of the human skin reveal age-related loss of fibroblast priming. *Commun. Biol.* **3**, 188 (2020).
