# Supplementary material for: Functionally distinct cancer-associated fibroblast subpopulations establish a tumor promoting environment in squamous cell carcinoma
Source: Nat Commun. 2023 Sep 5;14:5413. doi: 10.1038/s41467-023-41141-9 (PMC10480447; doi:10.1038/s41467-023-41141-9)
Supplement: Supplementary file 2 — Reporting Summary [file 41467_2023_41141_MOESM2_ESM.pdf]

nature portfolio

Corresponding author(s): Drs. Manuel Rodríguez-Paredes & Frank Lyko

Last updated by author(s): Aug 10, 2023

Reporting Summary

Nature Portfolio wishes to improve the reproducibility of the work that we publish. This form provides structure for consistency and transparency in reporting. For further information on Nature Portfolio policies, see our Editorial Policies and the Editorial Policy Checklist.

Statistics

For all statistical analyses, confirm that the following items are present in the figure legend, table legend, main text, or Methods section.

n/a	Confirmed	
☐	☒	The exact sample size (*n*) for each experimental group/condition, given as a discrete number and unit of measurement
☐	☒	A statement on whether measurements were taken from distinct samples or whether the same sample was measured repeatedly
☐	☒	The statistical test(s) used AND whether they are one- or two-sided<br>*Only common tests should be described solely by name; describe more complex techniques in the Methods section.*
☐	☒	A description of all covariates tested
☐	☒	A description of any assumptions or corrections, such as tests of normality and adjustment for multiple comparisons
☐	☒	A full description of the statistical parameters including central tendency (e.g. means) or other basic estimates (e.g. regression coefficient) AND variation (e.g. standard deviation) or associated estimates of uncertainty (e.g. confidence intervals)
☐	☒	For null hypothesis testing, the test statistic (e.g. *F*, *t*, *r*) with confidence intervals, effect sizes, degrees of freedom and *P* value noted<br>*Give P values as exact values whenever suitable.*
☒	☐	For Bayesian analysis, information on the choice of priors and Markov chain Monte Carlo settings
☒	☐	For hierarchical and complex designs, identification of the appropriate level for tests and full reporting of outcomes
☒	☐	Estimates of effect sizes (e.g. Cohen's *d*, Pearson's *r*), indicating how they were calculated

Our web collection on statistics for biologists contains articles on many of the points above.

Software and code

Policy information about availability of computer code

Data collection	For data collection no software was used.
Data analysis	For the analysis of single-cell RNA sequencing data the softwares CellRanger v4.0.0 and R v4.0.3, as well as the R packages Seurat v4.0.5, CellChat v1.4, InferCNV v1.6, CopyKAT v1.1, Slingshot v1.8, LIANA v0.1.11, and GSEA v4.3.2 were used.<br>For processing and analysis of RNA FISH images the softwares QuPath v0.3.2, Fiji v2.3.0 and the HiPlex Image Registration Software v2.0.1 from Bio-Techne were used. For statistical analyses the software SigmaPlot v14.0 was used.

For manuscripts utilizing custom algorithms or software that are central to the research but not yet described in published literature, software must be made available to editors and reviewers. We strongly encourage code deposition in a community repository (e.g. GitHub). See the Nature Portfolio guidelines for submitting code & software for further information.

Data

Policy information about availability of data

All manuscripts must include a data availability statement. This statement should provide the following information, where applicable:

- Accession codes, unique identifiers, or web links for publicly available datasets
- A description of any restrictions on data availability
- For clinical datasets or third party data, please ensure that the statement adheres to our policy

The scRNA-seq data generated in this study have been deposited in the Gene Expression Omnibus (GEO) database under accession code GSE218170. The scRNA-seq

publicly available data used in this study are available in the GEO database under accession codes GSE130973, GSE181907 and GSE141526. The GO and GSEA publicly available data used in this study are available in the DAVID Bioinformatics Database v6.8 and Molecular Signatures Database v2023.1.Hs, respectively. The remaining data are available within the Article, Supplementary Information or Source Data file.

Research involving human participants, their data, or biological material

Policy information about studies with human participants or human data. See also policy information about sex, gender (identity/presentation), and sexual orientation and race, ethnicity and racism.

Reporting on sex and gender	All 14 single-cell RNA sequencing samples and all 14 RNA FISH samples were taken from male donors, as this group constitutes the classical majority of patients. Sex was determined based on self-reporting.
Reporting on race, ethnicity, or other socially relevant groupings	All 14 single-cell RNA sequencing samples and all 14 RNA FISH samples were taken from fair-skinned donors, as keratinocyte carcinomas are the most common cancers in the fair-skinned population.
Population characteristics	All donors were between 47 and 93 years old. The samples from nine donors (six for single-cell RNA sequencing and three for RNA FISH) were classified as healthy skin. The samples from three donors (RNA FISH) were diagnosed with actinic keratosis. Six samples from five donors (three for single-cell RNA sequencing from two donors and three for RNA FISH) were diagnosed with Bowen's disease. The samples from ten donors (five for single-cell RNA sequencing and five for RNA FISH) were diagnosed with cutaneous squamous cell carcinoma.
Recruitment	Only participants with the following characteristics were recruited: male, older than 40 years, Fitzpatrick skin type I-III, and biopsy will be removed from an UV-exposed body part. All skin specimens were obtained from remnant tissue, not required for diagnostic purposes, of participants undergoing routine surgery at the Department of Dermatology, University Hospital of Heidelberg, after written informed consent by the participant. No participant compensation was provided. This restricted participant recruitment was required to obtain a homogeneous dataset but also limits the possibility to draw conclusions for other participant groups.
Ethics oversight	The study was approved by the Ethics Committee of Heidelberg University (S-091/2011) in compliance with the current legislation and institutional guidelines.

Note that full information on the approval of the study protocol must also be provided in the manuscript.

Field-specific reporting

Please select the one below that is the best fit for your research. If you are not sure, read the appropriate sections before making your selection.

[x] Life sciences　[ ] Behavioural & social sciences　[ ] Ecological, evolutionary & environmental sciences

For a reference copy of the document with all sections, see nature.com/documents/nr-reporting-summary-flat.pdf

Life sciences study design

All studies must disclose on these points even when the disclosure is negative.

Sample size	No sample size calculation was performed but minimum three samples per condition were chosen to achieve statistical significance.
Data exclusions	No data were excluded.
Replication	Single-cell RNA sequencing of UV-exposed healthy skin samples, Bowen's disease samples, and cutaneous squamous cell carcinoma samples was performed three, three, and five times, respectively. All replications were successful and conclusions are based on all samples. Multiplexed RNA FISH assays for general CAF detection with UV-exposed healthy skin samples, actinic keratosis samples, Bowen's disease samples, and cutaneous squamous cell carcinoma samples were performed three times each for all sample types. Multiplexed RNA FISH assays for detection of CAF interactions with cutaneous squamous cell carcinoma samples were performed two times. All replications were successful and conclusions are based on all samples.
Randomization	All samples were grouped according to histological diagnoses (healthy, actinic keratosis, Bowen's disease, cutaneous squamous cell carcinoma) performed by a dermatohistopathologist and/or a pathologist. All diagnoses were performed following current guidelines (Wolff et al., 2011) and are based on histological characteristics of epidermal keratinocytes.
Blinding	Blinding was not relevant for our study, as knowing the pathological diagnosis of each sample was essential for data analysis.

Reporting for specific materials, systems and methods

We require information from authors about some types of materials, experimental systems and methods used in many studies. Here, indicate whether each material, system or method listed is relevant to your study. If you are not sure if a list item applies to your research, read the appropriate section before selecting a response.

Materials & experimental systems

n/a	Involved in the study
☒	☐ Antibodies
☒	☐ Eukaryotic cell lines
☒	☐ Palaeontology and archaeology
☒	☐ Animals and other organisms
☒	☐ Clinical data
☒	☐ Dual use research of concern
☒	☐ Plants

Methods

n/a	Involved in the study
☒	☐ ChIP-seq
☒	☐ Flow cytometry
☒	☐ MRI-based neuroimaging
