# Supplementary material for: Functionally distinct cancer-associated fibroblast subpopulations establish a tumor promoting environment in squamous cell carcinoma
Source: Nat Commun. 2023 Sep 5;14:5413. doi: 10.1038/s41467-023-41141-9 (PMC10480447; doi:10.1038/s41467-023-41141-9)
Supplement: Supplementary file 10 — Description of Additional Supplementary Files [file 41467_2023_41141_MOESM10_ESM.pdf]

Title: Supplementary Data 1

Description: Gene expression in each cluster of all integrated scRNA-seq samples

Title: Supplementary Data 2

Description: Gene expression in each fibroblast cluster compared to all other fibroblasts

Title: Supplementary Data 3

Description: Gene expression in each CAF cluster.

Title: Supplementary Data 4

Description: Representative marker genes for cSCC-related CAFs.

Title: Supplementary Data 5

Description: Gene expression in each BCC fibroblast cluster.

Title: Supplementary Data 6

Description: Gene expression in each BCC CAF cluster (high expression of FAP, ACTA2 & cSCC-related CAF marker genes).
